# Pore performance: artificial nanoscale constructs that mimic the biomolecular transport of the nuclear pore complex

**DOI:** 10.1039/d2na00389a

**Published:** 2022-09-13

**Authors:** John Andersson, Justas Svirelis, Jesper Medin, Julia Järlebark, Rebekah Hailes, Andreas Dahlin

**Affiliations:** Department of Chemistry and Chemical Engineering, Chalmers University of Technology 41296 Gothenburg Sweden adahlin@chalmers.se

## Abstract

The nuclear pore complex is a nanoscale assembly that achieves shuttle-cargo transport of biomolecules: a certain cargo molecule can only pass the barrier if it is attached to a shuttle molecule. In this review we summarize the most important efforts aiming to reproduce this feature in artificial settings. This can be achieved by solid state nanopores that have been functionalized with the most important proteins found in the biological system. Alternatively, the nanopores are chemically modified with synthetic polymers. However, only a few studies have demonstrated a shuttle-cargo transport mechanism and due to cargo leakage, the selectivity is not comparable to that of the biological system. Other recent approaches are based on DNA origami, though biomolecule transport has not yet been studied with these. The highest selectivity has been achieved with macroscopic gels, but they are yet to be scaled down to nano-dimensions. It is concluded that although several interesting studies exist, we are still far from achieving selective and efficient artificial shuttle-cargo transport of biomolecules. Besides being of fundamental interest, such a system could be potentially useful in bioanalytical devices.

## Abstract 2

We review advances in artificial mimics of nuclear pore complexes, with particular focus on synthetic replication of the shuttle-cargo transport mechanism of biomolecules.

## Graphical abstract

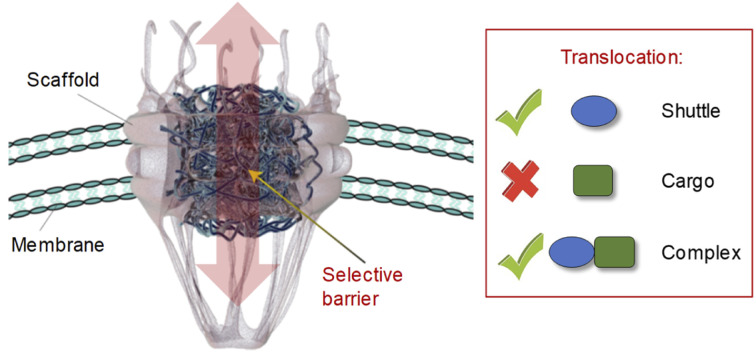

## Introduction and scope

**Fig. 1:**
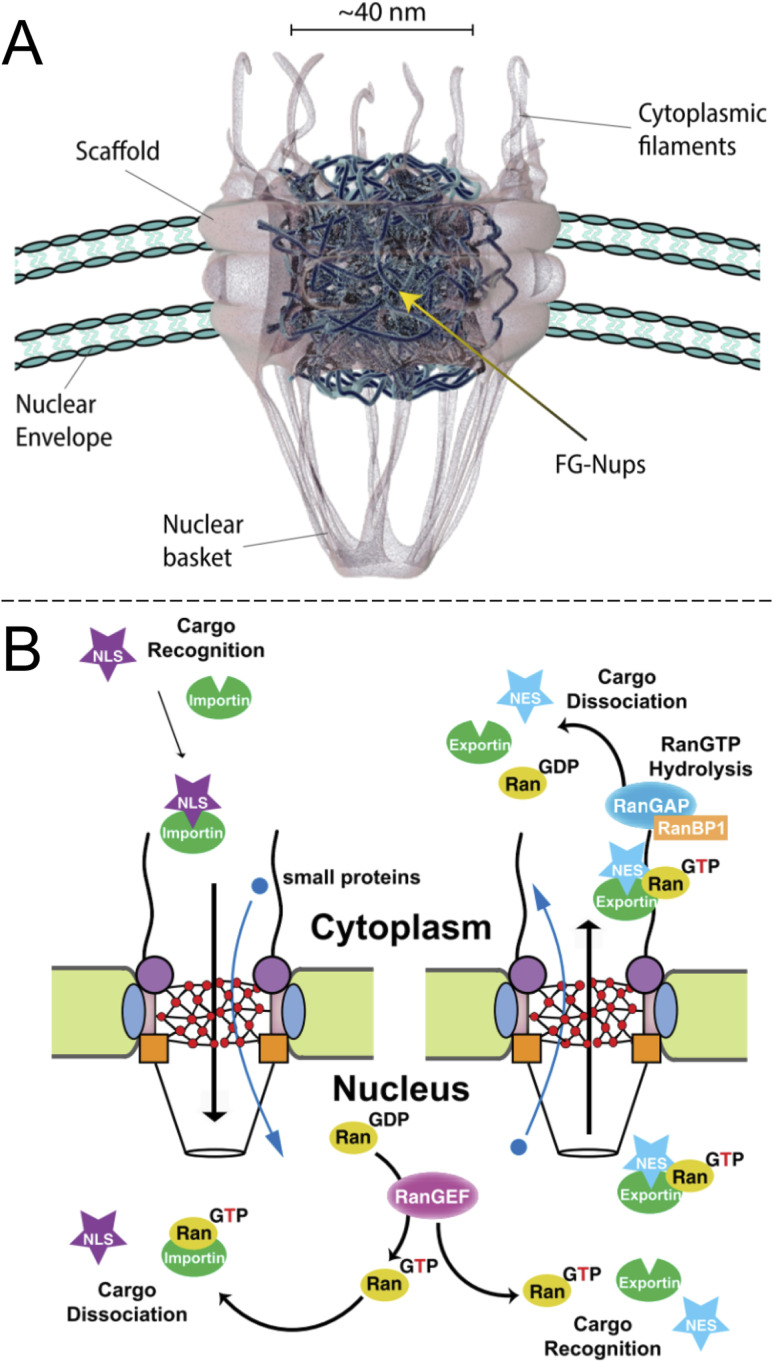
The biological system to be mimicked. (A) Drawing of the NPC with its main components (adapted with permission from Samir Patel, personal communication). (B) Principle of unidirectional nuclear import of proteins *via* RanGTP. Importin is the “shuttle” and NLS is the “cargo”. Reproduced with permission (CC BY) from Xylourgidis *et al.*^6^

Inside the envelope membrane surrounding the cell nucleus there exists a macromolecular machinery (Fig. 1A) that has fascinated molecular biologists and biophysicists alike since long ago. This assembly of multiple proteins, known as nucleoporins (Nups), forms the nuclear pore complex (NPC). The NPC has a total molecular weight as high as 125 Mg mol^−1^ (in vertebrates) and is generally well characterized.^1,2^ The most central functions of NPCs are to regulate access of proteins to the interior of the nucleus and to export mRNA. Although small molecules and ions diffuse freely through the barrier, molecules larger than ∼40 kg mol^−1^ can rarely pass unless they are bound to a transport protein, which thereby acts as a “shuttle” carrying a “cargo”. In other words, NPC transport occurs by a shuttle-cargo mechanism (Fig. 1B) and has quite remarkable selectivity. Artificially introduced cargoes as large as ∼39 nm, close to the inner NPC diameter, can be transported if they carry the right recognition sequence.^3^ Different shuttle proteins exist as well as different nuclear localization signals,^4^*i.e.* protein domains that bind the cargo to the shuttles.

Importantly, while the movement of the shuttle proteins is bidirectional, transport of cargo proteins is normally unidirectional (nuclear import) and thus energy consuming. This is possible because of specific conjugation to shuttles on one side of the NPC and release of cargo on the other, all regulated by sophisticated mechanisms.^5,6^ Specifically, the release of cargo inside the nucleus is associated with irreversible guanosine triphosphate hydrolysis to guanosine diphosphate and the transport cycle is completed by additional proteins.^7^ RNA is exported from the nucleus by similar processes^8,9^ and the dysregulation of the export proteins and associated receptors has been linked to cancer.^10^ However, the NPC construct itself is a passive barrier that provides facilitated diffusion.^7,11^ This raises the question if one can, perhaps in a relatively simple manner as no energy input is needed, construct an artificial system capable of shuttle-cargo transport. In other words, is it possible, in a synthetic manner, to achieve movement of cargo biomolecules only when they are bound to a shuttle, which moves freely through the pore construct? Unidirectionality could then be implemented in later developments given that the desired selective barrier properties are established with respect to the shuttle and cargo. As will be discussed, research has not yet progressed very far towards this goal and the task is very challenging both scientifically and technically. However, there are many reports that achieve important milestones and provide interesting insights.

Based on decades of research on NPC function, one can argue that the main reason why transport mechanisms are so difficult to understand is that the centre channel consists of Nups with intrinsic disorder,^12^ which occupy an average volume fraction of ∼20% throughout the interior. These contain characteristic phenylalanine–glycine (FG) motif repeats, which together form a barrier with a largely undefined and fluctuating structure of FG-Nups, although interactions do occur between the phenylalanines.^13^ In contrast, the rest of the NPCs have had the structures of their proteins determined in great detail.^2^ Many thorough studies have been performed from a biological starting point (a “top down” approach) in order to deduce more information about NPC transport. For instance, it is possible to selectively delete genes for certain FG-Nups, which eventually leads to failure of the native transport mechanisms and cell death.^14^ Physical tools such as high-speed atomic force microscopy,^15–17^ scanning electrochemical microscopy^18^ and single molecule fluorescence microscopy^8,19^ have also been used to directly probe “living” NPCs (often in isolated nuclei). Additionally, artificially designed constructs (non-proteins) capable of translocating NPCs in living cells have been demonstrated.^20^

In this review, the broader picture of shuttle-cargo transport is in focus and the purpose is not necessarily to understand the NPC specifically. Rather, we want to address the general question: to what extent is it possible to achieve shuttle-cargo transport in a synthetic manner, using “bottom up” strategies? We focus on the physical/chemical picture instead of living systems. From this perspective, the NPC becomes mostly a source of inspiration because it shows that shuttle-cargo transport is indeed possible. Furthermore, we note the many interesting predictions from theoretical studies, which have addressed the NPC specifically^7^ as well as the more general topic of disordered grafted macromolecules in nanopore geometries.^21^ Selected studies will be referred to, but our aim is not to cover all theoretical work. Instead, focus is on experimental research that attempts to construct functioning artificial transport systems.

It should be noted that chemically modified solid state nanopores have been reviewed previously on several occasions.^22–26^ Our goal is to dig further into NPC mimics specifically and the challenges associated with achieving shuttle-cargo transport. Systems that exhibit active gating mechanisms, *i.e.* pores that are either open or closed, may be relevant but are not the main focus as this is a quite different concept. Furthermore, purely size-selective systems such as unmodified porous membranes will be ignored since our interest lies with systems where the chemical modifications provide selectivity. In fact, even chemically modified nanopores may be irrelevant for this review if they are not functionalized with molecules that exhibit intrinsic disorder like FG-Nups, *i.e.* the grafted molecules should be some form of reasonably long polymeric chains. (To be precise, the contour length should be much larger than the persistence length.) For instance, a thin and dense organic coating may effectively reduce the diameter of a nanopore and thereby influence how large molecules can translocate,^25^ but such systems do not provide a transport mechanism with chemical selectivity. Furthermore, we do not cover the topic of very small pores (<10 nm) such as nanotubes *etc.* as these are often attempts to mimic selective ion permeability^23,24,26^ rather than macromolecular transport. However, bioinspired gels that exhibit selective transport^27^ will be discussed if they relate to the NPC. (Note that although such constructs are macroscopic, the effective pore sizes and cross-link densities are nanoscale features.) The scope of this review and the work described herein is mostly related to fundamental aspects of soft matter and whether we can increase our understanding of complex systems (the NPC and others) by studying simpler artificial mimics. Still, possible applications of a functioning artificial shuttle-cargo transport system will be briefly mentioned.

## FG-Nups on solid state nanopores

Solid state nanopores are of great interest in designing NPC mimics since they provide a stable structural foundation that also comes with single molecule sensing capabilities.^28^ Thiol chemistry on gold and silanization of silica are among the most widely used strategies for chemical functionalization.^25^ A straightforward strategy for designing artificial NPCs, which has indeed been relatively popular, is the grafting of FG-Nups obtained from biological sources, to the inner walls of solid state nanopores. Translocation events of individual transport proteins can then be detected by the ionic current (essentially a patch clamp measurement) and compared with the event frequency for another water-soluble protein, typically bovine serum albumin (BSA). Optical detection of the molecules that move through the pores, using fluorescent labels if needed, can also be implemented, often providing a total flux of molecules through multiple pores.

**Fig. 2:**
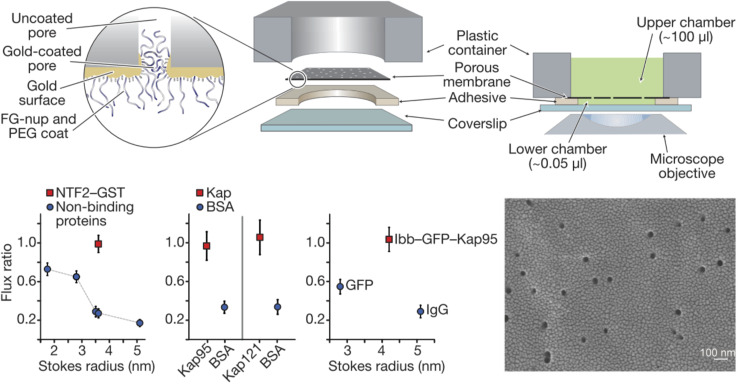
Pioneering work on solid state nanopores functionalized with FG-Nups. 6 μm thick polycarbonate membranes were covered with a thin gold layer on one side to which FG-Nups were grafted. The passive diffusive flux of proteins was measured and normalized to that measured for a control membrane with a thin oligo(ethylene glycol) coating. (A flux ratio of one essentially means the FG-Nups did not hinder transport.) The human nuclear transport factor 2 glutathione S-transferase (NTF2-GST) can translocate unhindered while the flux of other proteins is reduced. Similarly, karyopherins (Kap95 and Kap121), which are another class of transporters, translocate more frequently than BSA. Also, a shuttle-cargo complex consisting of Kap95 and green fluorescent protein (GFP) with the importin-β-binding domain (Ibb) translocated more frequently than GFP alone or an IgG antibody. The electron microscopy image shows the gold coated membrane before any chemical modifications. Reproduced with permission from Jovanovic-Talisman *et al.*^29^ Copyright 2009 Springer Nature.

Historically, the first study of nanopores functionalized with FG-Nups is that reported by Jovanovic-Talisman *et al.*^29^ who used a device containing two chambers separated by a polycarbonate membrane containing 30 nm diameter nanopores coated with a 15 nm gold film on one side, functionalized with either Nsp1 or Nup100 (Fig. 2). The FG-Nups were attached to the gold surface *via* thiol bonds from a single cysteine residue introduced by genetic engineering on the C-terminal end of the proteins,^30^ with a resulting grafting density estimated to 0.05 and 0.04 nm^−2^ for Nsp1 and Nup100, respectively. Fluorescently labelled human nuclear transport factor NTF2-GST as well as transport factors Kap95 and Kap121 were tested in comparison to green fluorescent protein (GFP), BSA and immunoglobulin G (IgG) as control proteins. The transportation was measured by fluorescence and compared with the diffusive flux through open pores. The transport proteins translocated seemingly unimpeded by the FG-Nups and an approximate three-fold difference in permeability could be observed between transport and 40 kg mol^−1^ control proteins. Importantly, a doubled translocation rate of GFP bound to Kap95 through a nuclear localization “signal” (a protein domain) could also be observed in comparison with GFP alone, thereby demonstrating shuttle-cargo transport properties. However, all membranes were leaky to all proteins tested, *i.e.* no membrane was strongly blocking any protein. In fact, even pores modified with poly(ethylene glycol) (PEG) of high molecular weight (30 kg mol^−1^) showed only a small reduction in diffusive transport. (Later work has shown complete blockage of protein diffusion through larger nanopores functionalized with shorter PEG chains, see below.) Tentatively, this is because PEG as large as 30 kg mol^−1^ is actually difficult to graft to the surface.^31^ Another question that arises from this (pioneering and important) work concerns the heterogeneity of the pores in the polycarbonate membrane. Is the selectivity actually perfect for some pores and very poor for others? This can only be revealed by measurements on single pores.

**Fig. 3:**
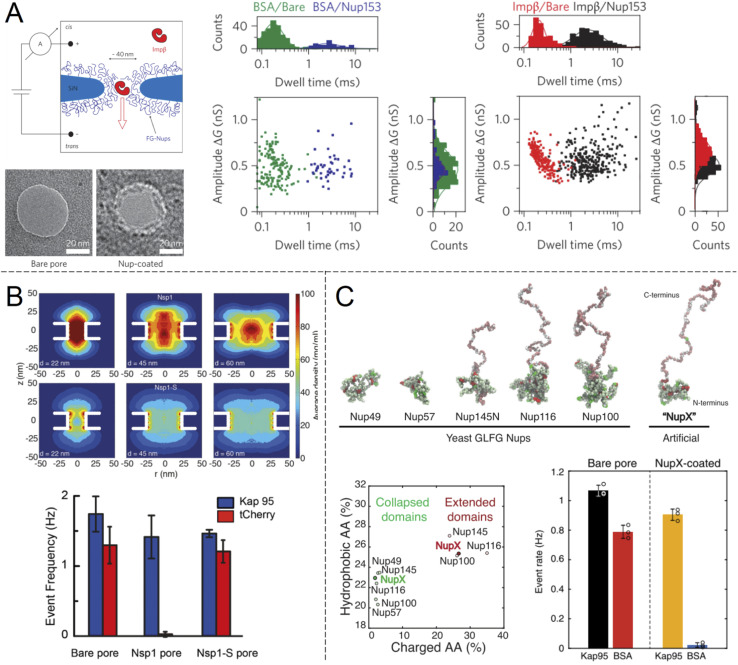
Work from the group of Dekker and co-workers showing ion current measurements of protein translocation through solid state nanopores with grafted FG-Nups. (A) First study of FG-Nup modified nanopores in silicon nitride. Transmission electron microscopy images show a pore before and after FG-Nup coating. The histograms show amplitudes and dwell times of single molecule translocation events of transport protein Impβ *vs.* BSA for bare and coated pores. Reproduced with permission from Kowalczyk *et al.*^32^ Copyright 2011 Springer Nature. (B) Similar work comparing native FG-Nup Nsp1 with the mutated less cohesive version Nsp1-S. The colormaps show the simulated peptide density profiles for different pore diameters. The bar plots show the translocation event frequency of the transport protein Kap95 *vs.* the control protein tCherry. Reproduced with permission (CC BY) from Ananth *et al.*^37^ (C) Similar work using a fully artificial FG-Nup sequence entitled NupX, inspired by native yeast FG-Nups. The collapsed and extended domains maintain similar degrees of charged and hydrophobic amino acids. The bar plots show the performance of NupX in terms of protein selectivity (Kap95 *vs.* BSA). Reproduced with permission (CC BY) from Fragasso *et al.*^40^

Selective translocation of the nuclear transport factor importin-β (Impβ) was demonstrated by Kowalczyk *et al.*^32^ using single solid state nanopores with 44 ± 2 nm diameter in 20 nm silicon nitride membranes functionalised with human Nup98 and Nup153 (Fig. 3A). The functionalization scheme utilized a triethoxysilane to first aminate the silicon nitride membrane, after which C-terminal cysteine modified FG-Nups were attached *via* a crosslinker that forms covalent bonds between amines and thiols, resulting in an FG-Nup grafting density of ∼0.02 nm^−2^. For the control protein BSA, which is similar in size and charge to Impβ, a decrease in the translocation frequency of 60 and 5 times could be observed for Nup98 and Nup153 coated pores respectively. In contrast, the translocation probability was barely affected at all for Impβ. The signal magnitudes (ion current change) were unaffected by the FG-Nups as expected, while the time of translocation was increased by more than an order of magnitude when the pores were coated by FG-Nups. Notably, this effect could be observed also for BSA, even though the total number of events were much fewer. In other words, even if BSA was unlikely to enter the FG-Nup network, it spent the same time inside it as Impβ. This may be viewed as surprising if one interprets a slow translocation as due to interactions with the polymer. However, we argue that one can also look at this in another way: if it is hard for the protein to find a way through the collection of chains, it will also lead to a delayed translocation time, even if there are no favourable interactions with the chains.

The many successful examples of the ‘FG-Nups + solid state pores’ approach, at least in terms of protein selectivity, confirm the biological importance of the disordered FG-Nups for selective barrier properties. Other nucleoporins essentially act as a fixed scaffold, though recent work suggests that the NPC diameter is somewhat dependent on the conditions that the cell experiences, such as osmotic stress.^33^ A relatively unexplored question is how the extremely strong electric field (∼10^7^ V m^−1^) inside the pore during ion current measurements affects transport, *i.e.* is the measurement itself really not influencing the translocation? However, Kowalczyk *et al.* also tested to change the voltage and saw no obvious effect on the translocation time, suggesting that this effect is not strong. Still, it should be noted that optical detection will not have this issue, but based on our literature survey it seems this approach has so far not been used for detecting individual molecules in solid state nanopores with FG-Nups. One reason could be that it is difficult to obtain a detectable fluorescent signal during the fast translocation events (∼1 ms). Here nanopores with metallic structures could play an important role by enhancing the optical signals.^34^

Another important question is to what extent the specific amino acid sequence of different FG-Nups influences the permeability of the barrier that forms inside the pore, with respect to transport proteins as well as others. Kowalczyk *et al.* already noted big differences in BSA translocation probability between orthologues Nup98 and Nup153.^32^ Specifically, the strength of multivalent inter- and intramolecular interactions of FG-Nups, commonly referred to as “cohesiveness”,^35^ has been shown to play a decisive role in the permeability. This is central for the selective phase model of NPC operation,^36^ where a higher degree of cohesiveness leads to a less permeable layer. The different degree of cohesion can be inferred from the amino acid sequence. In particular, phenylalanines give rise to hydrophobic interactions which make the network of disordered Nups denser.

In another single molecule solid state nanopore study, Ananth *et al.*^37^ investigated the role of the amino acid sequence and cohesive properties of the yeast FG-Nup called Nsp1 and a mutant “SG-Nup” version termed Nsp1-S (Fig. 3B). The mutated peptide, which still had a disordered structure and the same number of residues, was more hydrophilic and less cohesive because the phenylalanines were substituted with serines. Ion current measurements revealed a significantly lower conductance through nanopores coated with Nsp1 compared to Nsp1-S, suggesting that Nsp1 adopts a more compact morphology inside the pore due to its higher cohesiveness, which was also confirmed by coarse-grained simulations (one bead per amino acid). The grafting densities of Nsp1 and Nsp1-S were found to be very similar based on several results, including the conductance changes from free peptides.^37^ Detection of protein translocation events showed that selective protein transport was achieved with Nsp1 but not Nsp1-S. Remarkably, the translocation event frequency of the control protein (tCherry) through pores modified with Nsp1-S was the same as that of bare pores, while Nsp1 essentially fully blocked the passage.^37^ Thus, cohesiveness due to FG-repeats appears to be a crucial factor for selective transport in the native NPC. However, this does not mean that FG-repeats are an absolute prerequisite for constructing a selective shuttle-cargo transport system in a fully artificial setting, where one could potentially utilize other types of weak multivalent interactions associated with synthetic polymers. It should also be noted that, for native NPCs, large-scale protein sequence analysis^38^ and detailed simulations^39^ strongly suggest there are also electrostatic contributions to the transport selectivity.

Digging deeper into what determines the selective permeability of FG-Nups, a recent study by Fragasso *et al.*^40^ investigated the performance of an artificially designed FG-Nup (Fig. 3C). From a set of design principles based on averaged amino acid sequence information and physical properties of 5 naturally occurring FG-Nups with high cohesiveness, an artificial “NupX” was created, reflecting the characteristic cohesive and self-repulsive regions of the inspired natural FG-Nups, but featuring a very different amino acid sequence. The artificial NupX was evaluated in terms of its selective permeability to the transport protein Kap95 with the same methodology as in the other two studies.^32,37^ Indeed, Kap95 was found to translocate NupX coated nanopores with almost no hindrance compared to an uncoated pore, while the BSA control showed almost no translocation events after NupX functionalization, similar to tCherry for the case of Nsp1.^37^ Selectivity was confirmed with molecular dynamics simulations, which also revealed a similar type of pore-central density distribution as observed for Nsp1 (Fig. 3B). Overall, these findings strongly suggest that even though the FG-repeats are central for NPC function, both qualitatively and quantitatively, the exact amino acid sequence is not important. In a following study, Fragasso *et al.* also quantified the amount of transport proteins bound inside FG-Nup modified nanopores.^41^ The results showed that besides the fast translocation events, there is also a “slow phase” of more strongly interacting transport proteins. The authors discussed if and how the strongly bound proteins might contribute to the fast translocation of the loosely bound ones, which is a long-standing question in the NPC research field. Notably, it could be important to control the amount of bound shuttles (without cargo) for fast and efficient translocation of the shuttle-cargo also in artificial systems.

An alternative to ion current measurements is to use plasmonic nanopores, which also provide label-free detection (though rarely at the single molecule level) based on the local change in refractive index at the surface. Malekian *et al.*^42^ determined the affinity of Kapβ1 binding to FG-Nups inside plasmonic nanopore arrays with a metal-insulator-metal geometry. Using such structures, it is possible to selectively modify the pore interior by material-specific chemistry. However, nanoplasmonic measurements can only detect protein binding to the FG-Nups inside the pores, not their translocation through the pores. Furthermore, fabrication of pores with diameters approaching that of the NPC becomes quite challenging for a silicon nitride membrane sandwiched between two metal films. Malekian *et al.* managed to obtain data from samples with ∼60 nm pores. Unfortunately, the plasmonic activity is also very weak when the pore diameter becomes comparable to that of the NPC.^43^

## Solid state pores with other chemical modifications

Looking beyond FG-Nups, the concept of chemically modifying nanopores (or “nanochannels” in thicker membranes) to introduce transport selectivity is quite common in the literature. However, the number of studies is significantly reduced if one excludes studies of ion transport and rectification behaviour. Furthermore, relatively few articles have demonstrated selectivity with respect to another property than molecular size. This is a prerequisite for a shuttle-cargo transport system as the very same cargo should pass the barrier when conjugated to a shuttle, despite the fact that this will lead to an increase in size. Ku and Stroeve observed a relatively high selectivity between two different proteins attributed to differences in the isoelectric point.^44^ However, this required a lower salt content to enhance the electrostatic forces, which can generally be ignored at distances above the screening length (<1 nm at physiological salt). The approach of interest for this review is nanopores that are chemically modified such that they provide selectivity by molecular recognition, a feature which is less common in the literature.^23^ Although there has been recent interest in specific binding of analytes to the interior of nanopores, this has mostly been utilized for sensing applications rather than selective transport.^22^ Obviously, transport selectivity requires a coating that is at least comparable in thickness to the radius of the pore, otherwise all species will easily leak through an open centre channel. This is one reason why grafted polymer chains become interesting, in addition to their structural disorder and morphology changes,^21,23^ which can be thought of as mimicking FG-Nups.

**Fig. 4:**
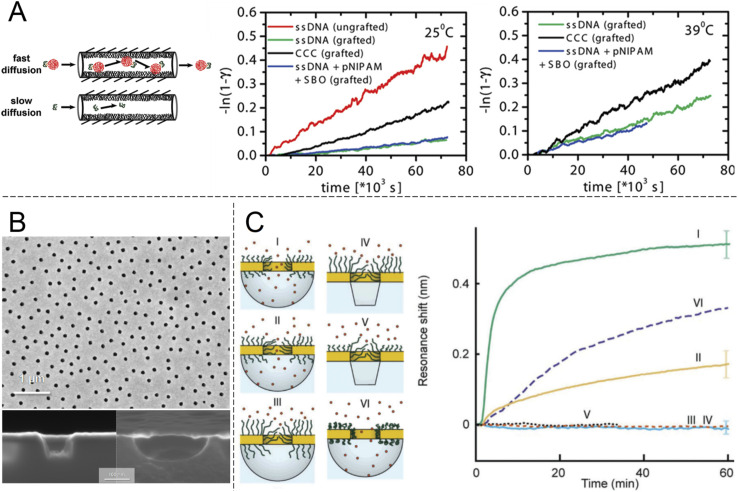
Nanopores modified with synthetic polymers. (A) Pioneering work by Caspi *et al.* on shuttle-cargo transport through an entirely synthetic system (the only biomolecule is the cargo). Nanopores with 50 nm nominal diameter in polycarbonate membranes, comparable to those in Fig. 2, were functionalized with PNIPAM. The cargo was also conjugated to PNIPAM in solution and the self-interactions of the polymer were suggested to be the cause for selective transport. The plots show transported amounts *vs.* time at different temperatures. Here “grafted” means PNIPAM-modified, CCC stands for the cargo carrier complex and SBO stands for the synthetic binding oligomer. Reproduced with permission from Caspi *et al.*^47^ Copyright 2008 American Chemical Society. (B) Electron microscopy images of plasmonic nanoscale cavities. Reproduced with permission (CC BY) from Malekian *et al.*^49^ (C) Sealing with PEG brushes prevents protein translocation as detected by the resonance shift (or lack thereof) due to protein adsorption in cavities underneath the pores in gold. The cavities have either vertical or curved surfaces depending on the etch method, as shown by the electron microscopy images. Pore sealing is achieved when the polymer is sufficiently long and the diameter sufficiently small. Reproduced with permission (CC BY) from Emilsson *et al.*^50^

Early work showed pores with some selectivity with respect to oligonucleotide sequences^45^ and even enantiomers,^46^ achieved by immobilizing receptors on the pore walls. Notably, the pores in such studies were made in relatively thick membranes (∼10 μm) consisting of track-etched polycarbonate or anodized alumina. Ultrathin silicon nitride membranes are normally needed to approach the NPC geometry (Fig. 3). Furthermore, the transport selectivity is typically not so high^45,46^ (less than an order of magnitude), potentially related to heterogeneity in the pore shapes. Nevertheless, the fact that chemical modifications can cause at least some selectivity by molecular recognition is intriguing. However, very few studies have actively aimed to achieve selective transport of other macromolecules (*i.e.* not ions) by grafting synthetic polymers (*i.e.* not FG-Nups). Here the work by Caspi *et al.*^47^ sticks out by explicitly aiming to reproduce NPC transport artificially (and predating even the work in Fig. 2). In this paper, nanopores were modified with poly(*N*-isopropylacrylamide) (PNIPAM) and the passive transport of fluorescently labelled single stranded DNA was measured (Fig. 4A). The concept of grafting PNIPAM chains to porous structures is well-known for creating membranes with thermo-responsive permeability,^48^ but the work by Caspi *et al.* instead shows a fully artificial shuttle-cargo transport mechanism. The only biological molecule was the cargo DNA, which was transported at least twice as fast through the functionalized nanopores when conjugated to PNIPAM in solution, despite the larger size of this conjugate. Interestingly, an increased transport rate of the shuttle-cargo complex compared to the pure cargo was observed even above the lower critical solution temperature of PNIPAM, where one would expect aggregation of all the polymer chains,^48^ whether they are grafted or free in solution. The authors generally attributed the effect to favourable hydrogen bond interactions between PNIPAM molecules. Unfortunately the PNIPAM amount inside the relatively long pores (6 μm) could not be characterized and can be expected to be very limited due to poor reactant access during polymerization,^48^*i.e.* much of the pore interior area could be bare polycarbonate.

An important criterion to improve selectivity is that polymer-functionalized nanopores are able to repel macromolecules in general. If a strong barrier is first obtained, one can then play around with the chemistry to introduce highly selective interactions with a potential shuttle construct. Hydrophilic and neutral (or zwitterionic) grafted polymers tend to be good at repelling other macromolecules, in particular proteins. This is well-known from decades of research struggling to create so called non-fouling interfaces for bioanalytical applications.^51^ However, measurements on planar surfaces are not always ideal for deducing the mechanism by which so called “polymer brushes”, which are obtained when chains stretch due to high grafting density, preventing protein adsorption. The brush may act as a barrier film that protects the underlying surface, but in principle adsorption can also be prevented by competitive polymer–surface interactions.^52^ Even for the extensively studied case of PEG brushes on gold (a common sensor surface material), the mechanism is still being debated and some work suggests it is competitive adsorption which prevents proteins from adsorbing.^53^ Using surface plasmon resonance, we^31^ and others^54^ have shown that PEG brushes can exclude other macromolecules (*e.g.* proteins or other polymers) from a zone near the surface that corresponds well to the expected brush thickness according to a de Gennes model,^50^ as well as the thicknesses obtained when measuring by other methods.^55^ These results confirm that the brushes act as barriers in a qualitative sense, but they cannot provide information about the protein distribution *vs.* the distance from the surface and the barrier strength in a quantitative manner. To address this question, our group systematically grafted PEG chains of different lengths to nanopores of different diameters in 30 nm thin gold films (Fig. 4B). Using the nanoplasmonic signal from the pore arrays, it was observed that as the planar surface brush height became comparable to the aperture radius, the pores entered a fully sealed regime with respect to serum proteins.^50^ Importantly, PEG is a homopolymer and has no cohesive nature. The degree of hydration for the brushes is above 80%^31,50,55^ and still they prevented protein translocation fully (within the uncertainty of the measurements). FG-Nups are different in their chemical nature compared to synthetic polymers, so this is not exactly contradictive, but still quite opposite to the results in Fig. 3B, where the less cohesive construct became leaky for proteins. In fact, the findings in Fig. 4C suggest that strong barriers can be created solely from entropic effects because the chains are not interacting and occur at a low volume fraction. In principle, one can imagine a state where proteins are present inside the brush and no bonds need to be formed or broken to reach this state. Still, this does not occur, which is consistent with the picture that the probability of finding a way through the (constantly moving) chains is too low. In thermodynamic terms, the conformational entropy loss of the chains due to the volume occupied by the proteins is too high. Favourable (enthalpic) interactions are then required to reduce the free energy penalty of insertion into a disordered polymer brush, as proposed long ago for the NPC.^56^ At the very least, the results in Fig. 4C prove that high cohesiveness among disordered polymer chains is not an absolute requirement for forming a strong barrier. Indeed, the importance of cohesiveness in the NPC continues to be debated. For instance, recent theoretical work by Gu *et al.*^35^ concluded that barrier strength is not always a monotonic function of the degree of cohesiveness and pointed out the important intrinsic connection with the density of the assembly. For a brush on a planar surface, the grafting density also influences polymer density. For a brush inside a pore, the molecular weight will also matter.

In a follow-up study on plasmonic nanopores, Emilsson *et al.*^57^ studied interactions between PEG brushes and IgG antibodies. It was shown that upon antibody binding the brush morphology changed considerably by local collapse of the PEG. Inside the pore, this effect made the brush barrier fully permeable for proteins again. Interestingly, an extremely low number of antibodies (probably just one) was sufficient to open each pore. This work can be viewed as an artificial analogy to the morphology changes observed in FG-Nup brushes (on planar surfaces) upon binding of transport proteins.^58^ However, while the PEG antibodies caused the brush barrier in the pores to “open”, this is not the case when transport proteins (the biological shuttles) bind to the FG-Nups in the NPC. In fact, they have been suggested to strengthen the barrier function in the so called Kap-centric model.^59^ Already in the work presented in Fig. 2, it was observed that the presence of transport proteins improved selectivity in the sense that the flux of BSA was reduced.^29^ This is supported by the fact that the mass of transport proteins inside the (yeast) NPC is almost as high as the mass of the FG-Nups themselves^2^ (not including the additional mass from cargo).

## Fully organic constructs

**Fig. 5:**
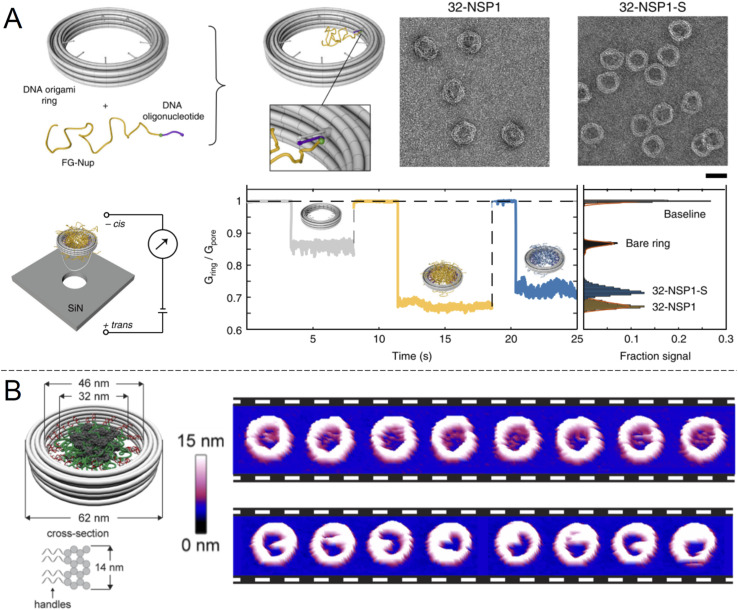
DNA origami strategies. (A) NPC-mimic with 34 nm inner diameter and 32 FG-Nups. Upon docking to a solid state nanopore, the conductance could be measured for Nsp1 and its mutant Nsp1-S. Scale bar is 50 nm. Reproduced with permission (CC BY) from Ketterer *et al.*^65^ (B) Another DNA origami construct with its dimensions indicated. The images show repeated AFM scans of the same constructs containing 48 copies of Nup100 (top) or Nsp1 (bottom). The time elapsed between each image is 1.6 s Reproduced with permission (CC BY) from Fisher *et al.*^66^ Copyright 2018 American Chemical Society.

An alternative to nanopores in inorganic materials is fully organic constructs, normally self-assembled in the solution phase. In particular, directed folding of DNA, so called “DNA origami”, has been used to build transmembrane pores for a decade, although initial pore architectures had just a few nm of inner diameter.^60^ Later developments showed somewhat larger DNA pores with size-selectivity in the range of typical proteins^61^ and electrical conductivity similar to or higher than the NPC.^62^ Also, several DNA pores that can be physically opened and closed by complementary sequences have been demonstrated.^61,63,64^ However, all of these constructs lacked the complexity required to even approach the selectivity of native NPCs. To do so, it was important to look beyond simply building a mimetic NPC of the correct dimensions but also control the number and spatial positioning of different FG-Nups. Ketterer *et al.*^65^ built an artificial NPC using a DNA origami ring with 34 nm inner diameter as a scaffold upon which to attach specific FG-Nups (Fig. 5A). The FG-Nups contained single strands complementary to free ends in the ring, enabling extremely precise control of the number and positioning of FG-Nups inside in comparison with all of the solid state nanopores described above. For instance, the 8-fold symmetry of FG-Nup positioning in the NPC^2^ can only be reproduced by DNA origami. As expected, FG-Nups displaying less hydrophobic interactions (the Nsp1-S mutant) formed less dense networks, as derived from the ion conductance. Simultaneously, Fischer *et al.*^66^ built another DNA origami pore scaffold comparable to the NPC with 46 nm inner diameter (Fig. 5B). FG-Nups were positioned either inside or outside the ring in specific numbers up to a maximum of 48, the number for budding yeast NPCs, leading to a different degree of crowdedness in the ring interior. Using multiple techniques, the authors also determined that the fine morphology of the assemblies was dependent on the exact FG-Nup sequence. (Nup100 and Nsp1 were compared, where the latter is less cohesive.) The authors also discussed important limitations of using AFM to probe the dynamics of the FG-Nup structure fluctuations,^66^ the main issue being that they may fluctuate in structure faster than the imaging can be performed. Yet, changes in the overall morphology of the ring interior detected over timescales of ∼1 s were attributed to real spontaneous fluctuations and not instrumental artifacts.

AFM has also been used in another study to observe the dynamics of FG-Nups inside a DNA origami NPC mimic and compare them with native NPCs.^16^ Such experiments revealed that FG-Nups in a DNA origami mimic interact collectively to form clumps that persist for ∼1 s, similarly to the results in Fig. 5B. Again, the exact behaviour differed subtly between types of FG-Nups. Comparatively, in native NPCs, FG-Nups appeared statically confined to a morphology, likely due to the presence of other macromolecular species in the living system^16^ (biological shuttles and cargo). These results provide important insights in the molecular dynamics of the NPC interior which can be important to consider when constructing artificial systems. However, it appears that to date no experiments showing selective protein translocation through DNA-based NPC mimics has been shown. One downside of DNA origami pores, similarly to native biological nanopores, is their need to be anchored somewhere. Chemical modifications that introduce hydrophobic groups allow spontaneous insertion into the membrane of lipid vesicles, but the vesicle interior is generally not easily accessible. A pore-spanning membrane is more suitable for access to both reservoirs,^60^ but such constructs are a bit more tedious to assemble. It is, however, possible to “dock” the entire DNA construct to a silicon nitride nanopore (Fig. 5A).

A few all-organic alternatives to DNA origami have been presented. Inspired by the NPC, Zhu *et al.*^67^ presented polymerosomes with selective microphase domains in the order of tens of nm that could efficiently mediate the transportation of macromolecules such as proteins and RNA across the vesicle membrane based on slight pH changes. Other organic nanopore constructs do exist and some are switchable,^68^ but again they tend to be limited to very small diameters (<10 nm) barely comparable to the NPC.

## Macroscopic gels

**Fig. 6:**
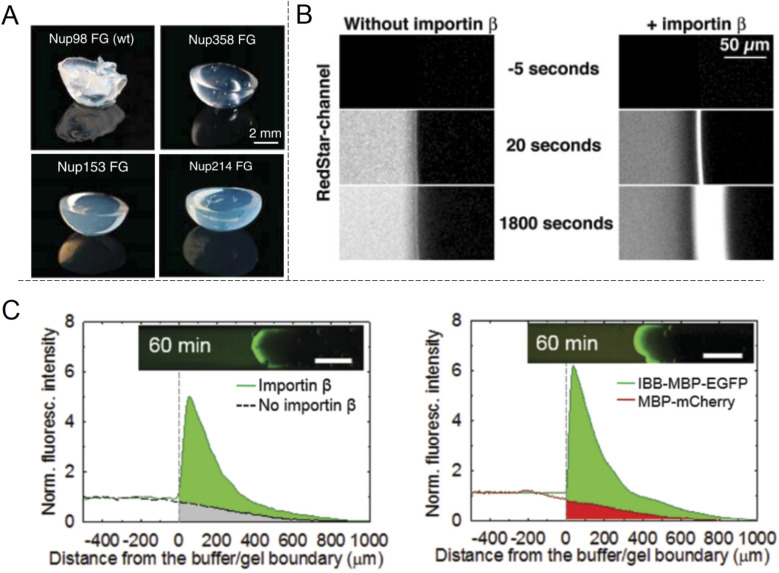
Selective protein transport inside macroscopic gels inspired by the NPC. (A) Photos showing gels spontaneously formed by different FG-Nup peptides. Reproduced with permission from Labokha *et al.*^71^ Copyright 2013 John Wiley and Sons. (B) Enhanced transport in a FG-hydrogel of a fluorescent fusion protein (acRedstar and the IBB domain) in the presence of Impβ. Reproduced with permission from Frey *et al.*^70^ Copyright 2007 Elsevier. (C) Example of shuttle-cargo transport through a gel prepared by an artificial peptide sequence. The fluorescence from a protein conjugate (IBB-MBP-EGFP) capable of binding to Impβ was measured in the presence or absence of Impβ (left plot). Additionally, a protein incapable of binding to Impβ (MBP-mCherry) was not transported as efficiently into the gel (right plot). This illustrates a shuttle-cargo transport mechanism. Reproduced with permission from Kim *et al.*^72^ Copyright 2015 John Wiley and Sons.

The shuttle-cargo transport of the NPC occurs inside a nanopore but can be viewed as a property associated with phases and local domains of the macromolecules in the nanoscopic interior. Hence, for the scope of this review, it can be relevant to look at selective protein diffusion also in macroscopic gels, given that hampered or enhanced motion is indeed an effect of attractive or repulsive interactions with the gel matrix and its nanoscale dynamic structure. It was shown in 2006 by Frey *et al.*^69^ that isolated FG-Nups could form hydrogels (Fig. 6A) and in 2007 that these gels exhibited selective protein transport.^70^ The results were interpreted in the framework of the ‘selective phase model’, where cohesiveness is essential and transport proteins move through the network by repeatedly breaking existing connections and attaching themselves to the hydrophobic FG domains instead. Importantly, it was also shown that a cargo protein could be transported >10^4^ times faster, in terms of the influx rate to the gel, when bound to a transporter (Fig. 6B). Although not directly comparable, this is a strong selectivity when considering what has been achieved with solid state nanopores^29,47^ as explained above.

Several following studies have used similar methodology and prepared NPC-inspired gels for measuring protein diffusivity within. Labokha *et al.*^71^ investigated 10 different gels formed by the FG-domains of 10 different Nups from Xenopus (clawed frog) and found that they exhibited very varying degrees of protein selectivity. For several of the gels, both a small (mCherry, 26 kg mol^−1^) and a large (tCherry, 105 kg mol^−1^) control protein were able to diffuse relatively quickly through the mesh and the larger protein even bound to a few of the gels.^71^ A nuclear transport protein (NTF2) showed strong preferential partitioning to all gels. In contrast to native Nups directly obtained from biological sources, work by Ader *et al.*^73^ used genetic engineering to obtain only parts of an FG-Nup (Nsp1p) for forming different hydrogels. Their behaviour was considerably different with respect to protein binding in comparison with gels formed by the full Nsp1p sequence. Following the direction of modified sequences, Kim *et al.* prepared two hydrogels consisting of a fully artificially engineered peptide sequence.^72^ In both cases, an amino acid sequence containing one FG was repeated 16 times to form the full peptide. Notably, this is not so different from the design principle used to prepare the “NupX” (Fig. 3C). Additionally, coiled domains were introduced at the ends to promote fast gelation. It was shown that a fluorescent model cargo protein diffused quicker through the artificial FG gel in the presence of Impβ (Fig. 6C), which recognizes a domain introduced on the cargo protein (again by genetic engineering). The enhanced transport was actually in part attributed to changes in the gel morphology upon incorporation of Impβ. Still, a non-cargo protein (that does not interact with Impβ) was not transported as efficiently (a factor of 3–5), even in the presence of Impβ. This once more shows a complete shuttle-cargo transport mechanism in NPC-inspired gels. Later, Kim *et al.* also proposed the concept of selective target capture inside synthetic FG-Nup gels for bioseparation purposes using the transport protein NTF2.^74^ There are also additional studies on NPC-inspired gels focusing on the gelation process and the fine structure.^75^ Notably, Celetti *et al.*^76^ showed that in microfluidic devices, FG-Nups can also phase separate into droplets, *i.e.* liquid-state assemblies with protein transport properties similar to the gels (which are solid in terms of rheology). Although still relatively large, such constructs can contribute to a better understanding of the NPC operation as wells as how to achieve shuttle-cargo transport artificially.

## Discussion: performance remains poor

Our overall impression after reviewing the literature is that many interesting studies exist, but an efficient artificial shuttle-cargo transport system for macromolecules is still quite far from realization. In fact, only two concrete examples have been found: reports by Caspi *et al.*^47^ (fully artificial system) and Johanovic-Talisman *et al.*^29^ (FG-Nups on solid state nanopores). We consider these studies pioneering, but the performance in terms of selectivity (primarily in terms of cargo *vs.* shuttle-cargo complex) is low, more precisely a factor between 2 and 5 (Fig. 2 and 4A). In both studies, the pores seemed leaky and cargo biomolecules passed through the nanopores only about twice as frequently when conjugated to shuttles.^29,47^ In contrast, using ion current measurements on single nanopores, efficient blocking of non-specific proteins while maintaining passage of transport proteins has been shown in a few cases,^37,40^ but there has been no demonstration of a complete shuttle-cargo transport mechanism. Clearly, further work is needed to match biological NPCs, which are a result of over one billion years of evolution.^2^ An impressively high selectivity has, however, been obtained with macroscopic gels, starting with the initial work by Frey *et al.*^70^ (Fig. 6B). Still, to truly resemble the NPC, these gels would have to be many orders of magnitude smaller or at least thinner. (In the work we have reviewed they do not even have an “exit” side.) It is not obvious how one would prepare “nanogels” that separate two compartments, nor how the transport selectivity would be affected by such miniaturization.

Why is it so hard to achieve the goal? Starting from scratch, one can identify some reasons why the task of constructing an artificial shuttle-cargo transport system is technically challenging. To begin with, the barrier must at least to some extent be able to change its morphology, for instance by being disordered in structure like a polymer brush. This is because any fully fixed structure will be almost solely a size filter and the shuttle-cargo complex (to pass through) will always be larger than the cargo (to be blocked). Even in the selective phase model the morphology is changed in the sense that the hydrophobic interactions are broken and reformed to allow proteins to pass.^36^ Yet the morphology changes are a delicate matter: if the barrier is disrupted entirely upon shuttle binding, the system loses its selectivity, *i.e.* it simply switches between open and closed states with respect to proteins.^57,64^ In other words, the shuttles must interact with the barrier and alter its morphology in a very particular manner. Furthermore, whatever effect causes repulsion between free cargo and the barrier will still be present when the shuttle-cargo complex translocates, *i.e.* the affinity to the shuttle must somehow dominate. At the same time, if the shuttles bind with too high affinity, they may not be spontaneously released again and the pore will likely be clogged.^70^ When considering all these logical criteria it is not surprising that it becomes challenging to construct a well-functioning artificial system, even with the tools that nanotechnology has provided in the last few decades. It should also be noted that transport efficiency in terms of flux is another important aspect besides selectivity and again the NPC is quite remarkable as it translocates hundreds of transport proteins simultaneously with a dwell time of only a few milliseconds for each.^7^ Indeed, inside the cell, the bottleneck in the full transport process does not seem to be the NPC passage, but the binding of cargo proteins to karyopherin shuttles.^9^

We also argue that another reason why there is still no high-performance artificial shuttle-cargo transport system for biomolecules is that we have a limited understanding of soft matter and intrinsically disordered macromolecules (in comparison with solid state materials). This is well-illustrated by decades of discussion about NPC operation and the large variety of the proposed models. It certainly does not help that experimentally; results that are almost contradicting can be found in the literature. For instance, we explained above how some studies show that a higher degree of cohesiveness of grafted FG-Nups is needed for strong barrier properties,^37^ while some other studies show that a highly hydrated “uncohesive” polymer brush, in the sense that it has no self-interactions, still can be an excellent barrier.^50^ Even if we gradually improve our understanding of barrier functions, the situation is further complicated by the change in morphology of disordered macromolecules as they interact with molecules in solution, an effect that is also sensitive to the nanoscale geometry.^77^ All in all it becomes very difficult to account for and measure all these effects, especially when considering their dynamics and non-equilibrium situations.

## Conclusion and outlook

We have reviewed artificial systems that mimic the nuclear pore complex in eukaryotic cells. The literature on this specific topic is relatively sparse compared to many other research fields, as evident from the bibliography. However, the topic is quite interdisciplinary and connects very well with highly popular research areas, such as solid state nanopore sensors and polymer brushes.

To summarize, ion current measurements have provided a lot of information on selective protein translocation through FG-Nups grafted to solid state nanopores. They have, however, not demonstrated a complete shuttle-cargo transport system. This has only been demonstrated with limited selectivity in two studies that measured passive diffusion. Some examples of NPC-inspired gels capable of shuttle-cargo transport can be found in the literature, but they are macroscopic. DNA origami has recently been used to make pores where FG-Nups can be positioned with extreme precision, but based on our literature survey no protein translocation experiments through such constructs have been presented to date.

Finally, it seems appropriate to ask the question why one would want to pursue the goal of constructing an artificial shuttle-cargo transport system. An obvious reason is that through studying artificial systems we can learn more about the biological nanomachinery. Artificial systems are easier to control and model as they contain very few components compared to the thousands of different proteins found inside a living cell alone. The behaviour observed in the artificial system can then be used to make educated guesses about how the biological system works. Unsurprisingly, understanding the NPC is critical for medical purposes.^5^ One can also think of applications of a functioning shuttle-cargo system in biotechnology. Novel protein separation methods have already been proposed.^74^ Specifically, one can envision that if the shuttle is also able to act as a receptor, it can capture specific targets from a complex sample and transport them to the other side of a membrane with nanopores. Such a feature could be of interest for future bioanalytical devices, though perhaps the main driving force for pursuing research on the topic of this review is scientific curiosity and a fascination for the complexity of soft matter.

## Conflicts of interest

There are no conflicts to declare.

## Supplementary Material
